# Genetic polymorphisms in human UDP-glucuronosyltransferases 1A7 and the risk of gastrointestinal carcinomas: A systematic review and network meta-analysis

**DOI:** 10.18632/oncotarget.18675

**Published:** 2017-06-27

**Authors:** Yingshi Zhang, Jun Hou, Fan Feng, Dandan Li, Qiyu Jiang, Xiaojuan Li, Qingchun Zhao, Bo-An Li

**Affiliations:** ^1^ Center for Clinical Laboratory, The 302nd Hospital of Chinese PLA, Beijing 100039, P.R. China; ^2^ Department of Pharmacy, General Hospital of Shenyang Military Area Command, Shenyang 110840, P.R. China; ^3^ Department of Clinical Pharmacy, Shenyang Pharmaceutical University, Shenyang 110016, P.R. China; ^4^ Research Center for Clinical and Transitional Medicine, The 302nd Hospital of Chinese PLA, Beijing 100039, P.R. China

**Keywords:** UDP-glucuronosyltransferases 1A7, polymorphism, gastrointestinal carcinoma, cancer, network meta-analysis

## Abstract

**Objective:**

To identify the association between gastrointestinal carcinomas (GIC) risk and UDP-glucuronosyltransferases (UGTs) 1A7 polymorphisms through a systematic review and network meta-analysis.

**Results:**

Seventeen studies were eligible, which included 7738 patients and 18 analyses. First, it was found that compared with non-cancer participants, UGT1A7*1 were significantly decreased in cancer patient groups, especially in hepatocellular carcinoma, colorectal carcinoma, and Asian population groups; UGT1A7*2 was significantly increased in hepatocellular carcinoma and Asian population groups; and UGT1A7*3 was significantly increased in hepatocellular carcinoma, colorectal carcinoma, Caucasian, and Asian population groups. Second, the UGT1A7 polymorphism alleles contrast model and the categorized UGT 1A7 genotypes were compared, and the outcomes revealed that the ratio of UGT1A7*3 vs *2 increased, which may indicate an increased risk for cancer, especially for the pancreatic carcinoma and Caucasian groups. The ratio of Intermediate vs Low increased as well, which may also indicate an increased risk for GIC.

**Materials and Methods:**

PubMed, Embase, and the Cochrane library were searched for publications up until May 2017. First, the UGT 1A7 gene polymorphisms genotype in GIC patients were compared with a non-cancer control group, and second, the UGT1A7 polymorphism alleles contrast model and UGT 1A7 genotypes categorized according to enzymatic activity were examined.

**Conclusions:**

There is a cancer risk associated with increased UGT1A7 *2 for the hepatocellular carcinoma and Asian groups and with increased UGT1A7 *3 for the hepatocellular carcinoma, colorectal carcinoma, Caucasian, and Asian groups. Moreover, in Caucasian patients with GIC, the ratio of UGT1A7 *3 vs *2 was increased.

## INTRODUCTION

Gastrointestinal carcinomas (GIC) present an increasing global public health threat, including several types of cancer, such as hepatocellular, colorectal, gastric, esophageal, and pancreatic cancer. GIC account for approximately 30% of all cancers worldwide, and most of them are characterized by a remarkable predominant incidence in males [1–2]. Hepatocellular carcinoma (HCC) is the fifth most common malignancy and the second leading cause of cancer-related deaths worldwide. The five-year survival rate is 15–17% [3]. Colorectal carcinoma (CRC) is the second leading cause of cancer-related deaths in the United States and the third most common malignant cancer worldwide [4]. Pancreatic carcinoma (PC) is the thirteenth most common cancer worldwide with mortality and morbidity are roughly the same [5].

For primary GIC with a hidden onset, rapid development, and a high degree of malignancy, an early clinical diagnosis is difficult. Many patients with symptomatic treatment are diagnosed in the late stages of cancer, and most have local metastasis and distant metastasis. GIC cancer is not sensitive to radiotherapy or chemotherapy, and a postoperative relapse is extremely common, which are the major reasons for a high mortality rate [6–7]. Although the progress of the various treatment modalities, including the surgical removal of tumors, has significantly improved the long-term survival for patients with GIC in recent years, the overall prognosis is still not optimistic. Previous research [8–9] has shown that the distribution of polymorphism variations of different forms of metabolic enzymes is related to cancer susceptibility, mainly due to metabolic enzymes in the body metabolism of carcinogenic substances.

Human UDP-glucuronosyltransferases (UGTs) are an enzyme superfamily, which could catalyze the glucuronidation of a diverse range of compounds, including endogenous metabolites (e.g., bilirubin and steroid hormones), therapeutic drugs, and various classes of chemical carcinogens (e.g., heterocyclic and polycyclic hydrocarbons and heterocyclic amines) [10–11]. Polymorphic alleles of UGT1A7 have been described: alleles *3 and *4 are associated with decreased enzyme activity, and allele *2 is associated with an activity similar to the wild-type allele *1 [12]. The UGT1A7 protein sequences differ at amino acid positions 129, 131, and 208. Various combinations create four distinct allelic variants in human populations: UGT1A7 *1 (N^129^R^131^W^208^), *2 (K^129^K^131^W^208^), *3 (K^129^K^131^R^208^), and *4 (N^129^R^131^R^208^). A haplotype analysis revealed that the polymorphisms at position 129 and 131 are in complete disequilibrium linkage, whereas the polymorphism at position 208 occurs independently. Based on these data, two polymorphisms (e.g., N^129^K and W^208^R) were detected for the UGT1A7 genotypic. UGT1A7 *3 and UGT1A7 *4 produce a lower corresponding catalytic activity for several substrates, including the B[a]P metabolites, when compared with the wild-type UGT1A7 *1 encoding enzyme (see Supplementary Table 1) [13].

Numerous valuable epidemiologic studies have suggested that UGT1A7 affects individual susceptibility to various carcinomas, such as HCC [14], CRC [15], and PC [16]; however, the observed results still require additional research to be confirmed. Recently, UGT1A7 mutant-type (especially *3 and *4) gene polymorphisms have been identified as having a high risk of cancer, while others did not yield significant results. Pairwise meta-analyses [17–18] have been conducted to identify the cancer risk of different genotypes; however, only direct evidence was considered, and whether the UGT1A7 polymorphisms are a risk factor for cancer susceptibility was not investigated. No previous reviews have provided a comprehensive overview with a network meta-analysis and meta-regression.

## RESULTS

### Systematic review and qualitative assessment

**Figure 1:**
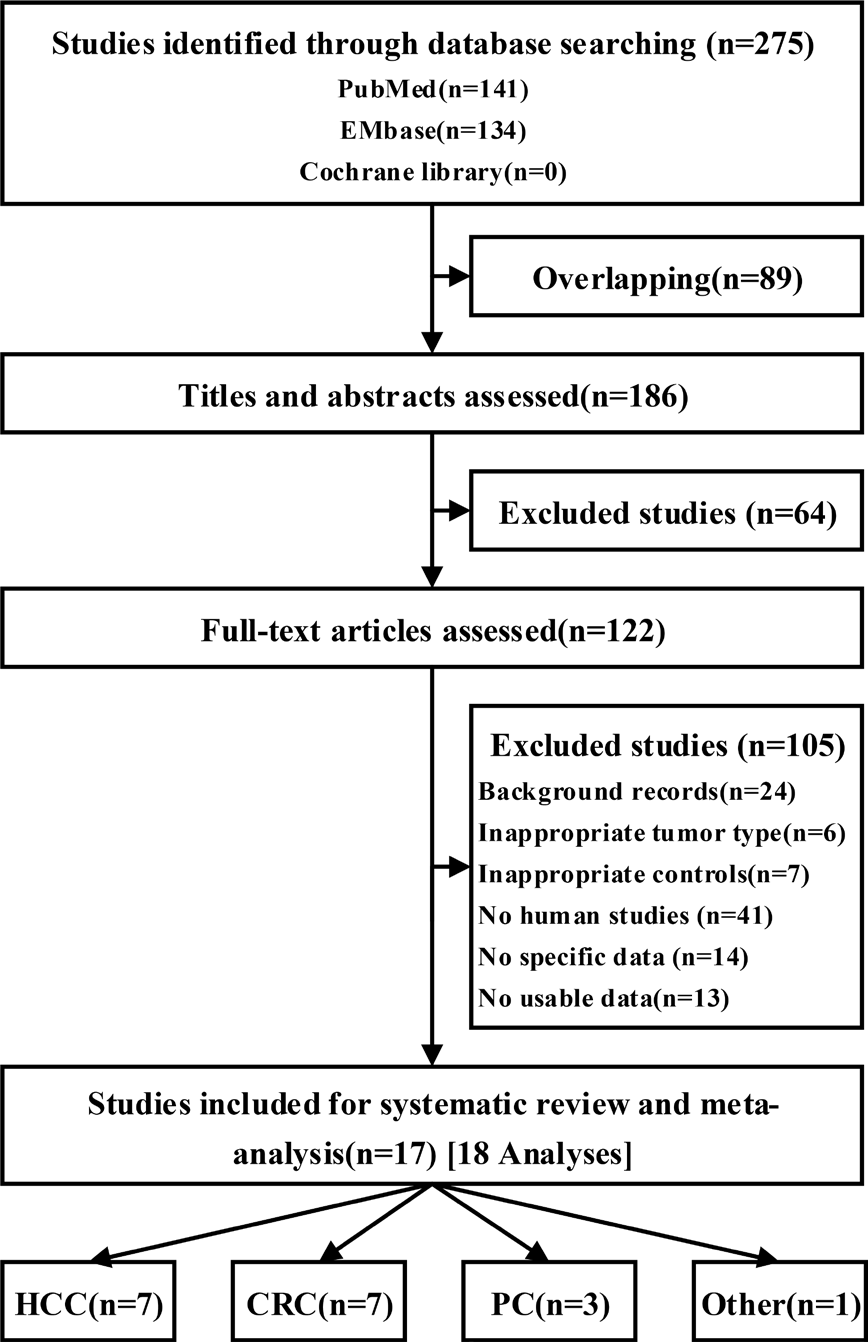
Flow of studies during the review process for the systematic review and meta-analysis

Overall, 186 unique citations were identified using the search strategy. A total of 17 [19–35] studies (198 analyses, *n* = 7738) and four UGT1A7 alleles were included (Figure 1). The mean study sample size was 455, ranging between 128 and 1645 participants. The publications from 2002 to 2012 were mostly conducted in Asia. In total, 2742 patients with GIC were assigned to the experiment group, and 4996 non-cancer participants were assigned to the control group. Seven trials on participants with HCC, 7 on CRC, 3 on PC, and 1 on proximal digestive tract cancer were examined.

Table 1 summarizes the differences in the fundamental characteristics between the experiment group and the control group (see full characteristics information in Supplementary Table 2). The statistics showed that the two groups had similar baseline results in age, smoking rate, alcohol rate and HCV infection rate;the GIC group had higher proportion of male and HBV infection rate. Because of there were differences in cancer type and ethnicity, substantial heterogeneity appeared in the baseline analyses. The assessments of study quality are presented in Supplementary Table 3, and the NOS scale score result shows that all included studies had an acceptable quality.

**Table 1:** Characteristics of baselines in subjects with or without gastrointestinal cancer

	Case vs. Control (OR, 95%CI)	Heterogeneity
**Age (year)**	0.60 (0.22∼0.99)*	*P* = 0.000, *I*^2^ = 97.5%
**Male**	1.33 (1.05∼1.68)	*P* = 0.000, *I*^2^ = 67.4%
**Smoking**	0.61 (0.36∼1.04)	*P* = 0.000, *I*^2^ = 80.3%
**Alcohol**	1.09 (0.81∼1.45)	*P* = 0.334, *I*^2^ = 10.9%
**HBV infection**	5.41 (1.38∼21.22)	*P* = 0.000, *I*^2^ = 93.2%
**HCV infection**	1.62 (0.89∼2.94)	*P* = 0.012, *I*^2^ = 69.1%

*Standardized mean difference; HBV, Hepatitis B Virus; HCV, Hepatitis B Virus.

### Pairwise meta-analysis– UGT1A7 alleles

**Figure 2:**
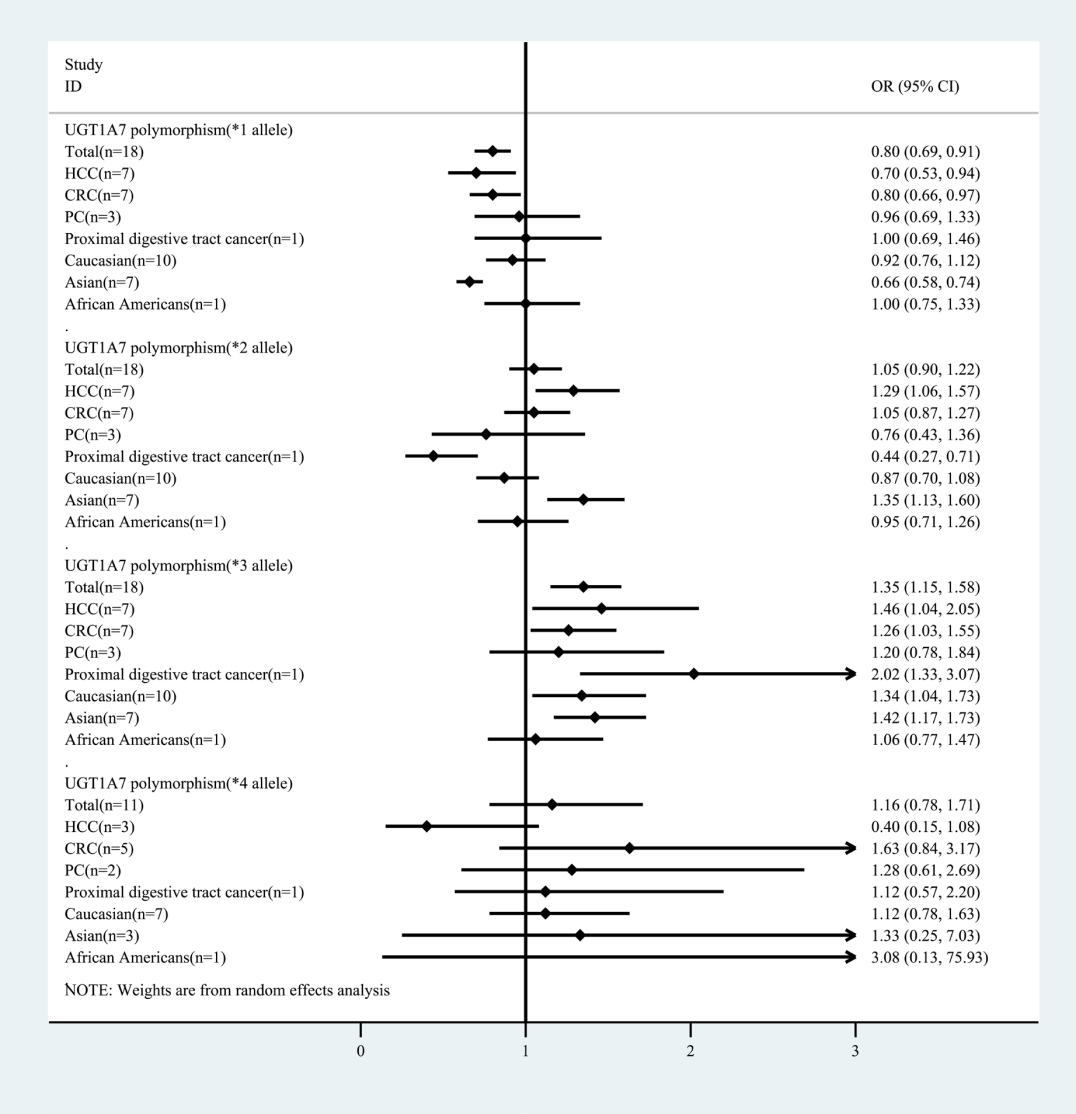
Summary of the meta-analysis of cancer risk associated with UGT1A7 polymorphism alleles (*1, *2, *3 and *4) stratified by cancer type and ethnicity

Figure 2 and Table 2 summarize the results of cancer risk associated with UGT1A7 polymorphism alleles (*1, *2, *3 and *4) stratified by cancer type and ethnicity. UGT1A7*1 was associated with a significant reduction in the GIC group compared with the control group (OR: 0.80, 95% CI: 0.69 to 0.91) and was accompanied by substantial heterogeneity (*P* = 0.000, *I*^2^ = 71.8%). The *P* value was calculated using a meta-regression, and the results revealed that cancer type (HCC, CRC, PC, and proximal digestive tract cancer) might influence heterogeneity, with *P* = 0.006. A similar effect size was found in the subgroups HCC, CRC, and Asian population. Little evidence of bias could be found by the Begg’s test and Egger’s test, with a low to moderate quality of evidence according to the GRADE assessment.

**Table 2:** Meta-regression and quality of evidence for the associations between UDT 1A7 polymorphic alleles vs. control on primary outcomes

UGT1A7 polymorphism	Participants (T/C)	Heterogeneity (*P*, *I*^2^)	Meta-regression (*P*)	Quality of evidence	Publication bias	
					Begg’s (*P*)	Egger’s (*P*)
*1						
Total^#^	2742/4996	*P* = 0.000, *I*^2^ = 71.8%		Low	*P* = 0.495	*P* = 0.584
Cancer types						
HCC(*n* ***=*** 7)^#^	997/1177	*P* = 0.000, *I*^2^ = 79.9%	*P* = 0.006*	Low	*P* = 0.453	*P* = 0.740
CRC(*n* ***=*** 7)^#^	895/1860	*P* = 0.005, *I*^2^ = 67.9%		Low	*P* = 0.453	*P* = 0.361
PC(*n* ***=*** 3)	349/1749	*P* = 0.005, *I*^2^ = 67.9%		Low	*P* = 0.117	*P* = 0.690
Proximal digestive tract cancer (*n* ***=*** 1)	76/210	-				
Ethnicity						
Caucasian (*n =* 10)	1351/3062	*P* = 0.001, *I*^2^ = 68.9%	*P* = 0.205	Low	*P* = 0.421	*P* = 0.651
Asian (*n =* 7)^#^	1194/1732	*P* = 0.292, *I*^2^ = 18.1%		Moderate	*P* = 0.051	*P* = 0.026
African Americans (*n =* 1)	197/202	-				
*2						
Total	2742/4996	*P* = 0.000, *I*^2^ = 68.7%		Low	*P* = 0.225	*P* = 0.434
Cancer types						
HCC (*n =* 7)^#^	997/1177	*P* = 0.124, *I*^2^ = 40.1%	*P* = 0.012*	Low	*P* = 0.881	*P* = 0.896
CRC (*n =* 7)	895/1860	*P* = 0.026, *I*^2^ = 58.1%		Low	*P* = 0.652	*P* = 0.696
PC (*n =* 3)	349/1749	*P* = 0.007, *I*^2^ = 80.1%		Low	*P* = 0.117	*P* = 0.001
Proximal digestive tract cancer (*n =* 1)^#^	76/210	-				
Ethnicity						
Caucasian	1351/3062	*P* = 0.000, *I*^2^ = 69.7%	*P* = 0.470	Low	*P* = 0.128	*P* = 0.147
Asian^#^	1194/1732	*P* = 0.141, *I*^2^ = 37.7%		Low	*P* = 0.881	*P* = 0.322
African Americans	197/202	-				
*3						
Total^#^	2742/4996	*P* = 0.000, *I*^2^ = 75.0%		Low	*P* = 0.019	*P* = 0.004
Cancer types						
HCC (*n =* 7)^#^	997/1177	*P* = 0.000, *I*^2^ = 81.5%	*P* = 0.039*	Low	*P* = 0.293	*P* = 0.100
CRC (*n =* 7)^#^	895/1860	*P* = 0.006, *I*^2^ = 66.6%		Low	*P* = 0.293	*P* = 0.222
PC (*n =* 3)	349/1749	*P* = 0.017, *I*^2^ = 75.5%		Low	*P* = 0.117	*P* = 0.484
Proximal digestive tract cancer (*n =* 1)^#^	76/210	-				
Ethnicity						
Caucasian^#^	1351/3062	*P* = 0.000, *I*^2^ = 81.5%	*P* = 0.023*	Low	*P* = 0.025	*P* = 0.028
Asian^#^	1194/1732	*P* = 0.041, *I*^2^ = 54.4%		Low	*P* = 0.881	*P* = 0.353
African Americans	197/202	-				
*4						
Total	1110/1869	*P* = 0.289, *I*^2^ = 16.3%		Moderate	*P* = 1.000	*P* = 0.656
Cancer types						
HCC (*n =* 3)	236/305	*P* = 0.543, *I*^2^ = 0.0%	*P* = 0.951	Moderate	*P* = 0.602	*P* = 0.625
CRC (*n =* 5)	685/1014	*P* = 0.272, *I*^2^ = 22.3%		Low	*P* = 0.624	*P* = 0.634
PC (*n =* 2)	113/340	*P* = 0.828, *I*^2^ = 0.0%		Low	*P* = 0.317	-
Proximal digestive tract cancer (*n =* 1)	76/210	-				
Ethnicity						
Caucasian (*n =* 7)	584/1117	*P* = 0.862, *I*^2^ = 0.0%	*P* = 0.598	Moderate	*P* = 0.099	*P* = 0.076
Asian (*n =* 3)	329/550	*P* = 0.012, *I*^2^ = 77.6%		Very Low	*P* = 0.602	*P* = 0.989
African Americans (*n =* 1)	197/202	-				

^#^Results with significant differences; *factors could be an important source of heterogeneity.

In addition, a significant increase was found only in the HCC group and Asian population group when UGT1A7*2 was compared in the cancer patient group and the control group. In general, an increasing trend was found in the GIC group (1.05, 0.90 to 1.22) accompanied by substantial heterogeneity (*P* = 0.000, *I*^2^ = 68.7%), while the different types of cancer could still be heterogeneous sources by meta-regression (*P* = 0.012). Little evidence of bias was found by the Begg’s test and Egger’s test, and there was low-quality evidence according to the GRADE assessment.

Moreover, the increase in UGT1A7*3 may be associated with a GIC risk (1.35, 1.15 to 1.58) with substantial heterogeneity (*P* = 0.000, *I*^2^ = 75.0%). Similar results were found in the subgroups of GCC, CRC, proximal digestive tract cancer, Caucasians, and Asians. The origin of heterogeneity could be different cancer types (*P* = 0.039) or ethnicities (*P* = 0.023) based on the meta-regression. Little evidence of bias was found by the Begg’s test and Egger’s test, and the evidence was low-quality according to the GRADE assessment.

Finally, UGT1A7*4, which may be associated with an increased risk of cancer (1.16, 0.78 to 1.71), was accompanied by low heterogeneity (*P* = 0.289, *I*^2^ = 16.3%). The meta-regression did not reveal the source of heterogeneity.

In general, the pairwise meta-analysis suggested that the UGT1A7*3 allele was a risk factor for GIC. In addition, this phenomenon was found to be more prominent in some cancer type subgroups, such as HCC and CRC, and some ethnic subgroups, such as Caucasian and Asian populations; however, this conclusion was not sufficiently systematic, and the meta-regression results also showed that the differences between the two groups varied (*P* < 0.05). Thus, further comparisons using pairwise meta-analyses and network meta-analyses between the UGT1A7 polymorphism alleles contrast model and the categorized UGT 1A7 genotypes are necessary.

### Pairwise meta-analysis and network meta-analysis

The evaluations of the UGT1A7 polymorphism alleles contrast model and the categorized UGT 1A7 genotypes are presented in Table 3. Supplementary Figure 1 provides the network weight of eligible comparisons, which also shows the available direct comparisons and the network of trials. For patients with HCC, a significant cancer risk resulted from UGTA7*3 vs *2 (pairwise: 1.24, 1.03 to 1.48) and Intermediate vs Low (pairwise: 2.55, 2.02 to 3.21; network: 5.10, 2.53 to 10.28). In addition, for patients with CRC, a significant cancer risk was found from UGTA7*3 vs *2 (pairwise: 1.55, 1.37 to 1.75) and Intermediate vs Low (pairwise: 5.33, 3.44 to 8.90; network: 19.15, 8.35 to 43.90). Moreover, for patients with PC, a significant cancer risk from UGTA7*3 vs *2 (pairwise: 1.94, 1.20 to 3.15; network: 2.58, 1.27 to 5.25) was identified.

**Table 3:** UGT1A7 polymorphism alleles contrast models and categorized UGT 1A7 genotypes outcomes: Comparisons of random effects pairwise meta-analysis with the network meta-analysis

Comparisons	Number of Studies with Direct Comparisons	Pairwise Meta-analysis OR (95% CI)	Heterogeneity (*P*, *I*^2^)	Network Meta-analysis OR (95% CrI)
**HCC**				
*2 vs *1	7 (997)	0.50 (0.40, 0.64)	*P* = 0.003, *I*^2^ = 70.3%	0.37 (0.23, 0.59)
*3 vs *1	7 (997)	0.65 (0.47, 0.90)	*P* = 0.000, *I*^2^ = 80.5%	0.37 (0.23, 0.59)
*4 vs *1	3 (236)	0.03 (0.01, 0.10)	*P* = 0.193, *I*^2^ = 39.2%	0.51 (0.32, 0.83)
*3 vs *2	7 (997)	1.24 (1.03, 1.48)*	*P* = 0.123, *I*^2^ = 39.6%	1.40 (0.86, 2.26)
*4 vs *2	3 (236)	0.05 (0.02, 0.12)	*P* = 0.843, *I*^2^ = 0.0%^#^	0.10 (0.04, 0.26)
*4 vs *3	3 (236)	0.04 (0.02, 0.09)	*P* = 0.616, *I*^2^ = 0.0%^#^	0.07 (0.03, 0.18)
Intermediate vs Low	7 (997)	2.55 (2.02, 3.21)*	*P* = 0.205, *I*^2^ = 29.3%	5.10 (2.53, 10.28)*
High vs Low	7 (997)	0.03 (0.01, 0.10)	*P* = 0.023, *I*^2^ = 59.0%	1.38 (0.68, 2.83)
High vs Intermediate	7 (997)	0.49 (0.33, 0.74)	*P* = 0.000, *I*^2^ = 78.9%	0.27 (0.13, 0.55)
**CRC**				
*2 vs *1	7 (1320)	0.62 (0.46, 0.83)	*P* = 0.003, *I*^2^ = 85.3%	0.48 (0.28, 0.81)
*3 vs *1	7 (1320)	0.72 (0.50, 1.05)	*P* = 0.000, *I*^2^ = 91.1%	0.60 (0.35, 1.01)
*4 vs *1	5 (685)	0.05 (0.01, 0.20)	*P* = 0.000, *I*^2^ = 90.3%	0.06 (0.03, 0.13)
*3 vs *2	7 (1320)	1.55 (1.37, 1.75)*	*P* = 0.000, *I*^2^ = 76.6%	1.26 (0.74, 2.13)
*4 vs *2	5 (685)	0.10 (0.03, 0.37)	*P* = 0.000, *I*^2^ = 91.4%	0.13 (0.06, 0.27)
*4 vs *3	5 (685)	0.06 (0.01, 0.24)	*P* = 0.000, *I*^2^ = 89.7%	0.10 (0.05, 0.22)
Intermediate vs Low	7 (1320)	5.33 (3.44, 8.90)*	*P* = 0.000, *I*^2^ = 84.4%	19.15 (8.35, 43.90)*
High vs Low	7 (1320)	0.78 (0.35, 1.73)	*P* = 0.000, *I*^2^ = 89.^2^%	0.79 (0.33, 1.87)
High vs Intermediate	7 (1320)	0.15 (0.09, 0.26)	*P* = 0.000, *I*^2^ = 85.7%	0.04 (0.02, 0.10)
**PC**				
*2 vs *1	3 (349)	0.52 (0.27, 0.99)	*P* = 0.005, *I*^2^ = 80.9%	0.41 (0.20, 0.83)
*3 vs *1	3 (349)	1.12 (0.92, 1.37)	*P* = 0.434, *I*^2^ = 0.0%^#^	1.05 (0.53, 2.09)
*4 vs *1	2 (113)	0.08 (0.00, 1.19)	*P* = 0.009, *I*^2^ = 85.^2^%	0.11 (0.04, 0.34)
*3 vs *2	3 (349)	1.94 (1.20, 3.15)*	*P* = 0.052, *I*^2^ = 66.1%	2.58 (1.27, 5.25)*
*4 vs *2	2 (113)	0.21 (0.03, 1.34)	*P* = 0.084, *I*^2^ = 66.6%	0.28 (0.10, 0.84)
*4 vs *3	2 (113)	0.08 (0.01, 1.39)	*P* = 0.007, *I*^2^ = 86.0%	0.11 (0.04, 0.32)
**Caucasian**				
*2 vs *1	9 (1275)	0.69 (0.56, 0.84)	*P* = 0.013, *I*^2^ = 58.9%	0.58 (0.44, 0.76)
*3 vs *1	9 (1275)	1.10 (0.98, 1.24)	*P* = 0.312, *I*^2^ = 14.6%	1.11 (0.85, 1.46)
*4 vs *1	6 (508)	0.08 (0.00, 1.19)	*P* = 0.000, *I*^2^ = 84.3%	0.12 (0.07, 0.20)
*3 vs *2	9 (1275)	1.55 (1.37, 1.75)*	*P* = 0.372, *I*^2^ = 7.6%	1.94 (1.47, 2.55)*
*4 vs *2	6 (508)	0.10 (0.03, 0.37)	*P* = 0.000, *I*^2^ = 85.0%	0.21 (0.12, 0.35)
*4 vs *3	6 (508)	0.06 (0.01, 0.24)	*P* = 0.000, *I*^2^ = 87.6%	0.11 (0.06, 0.18)
Intermediate vs Low	6 (927)	4.98 (3.56, 6.95)*	*P* = 0.067, *I*^2^ = 51.4%	14.79 (6.27, 34.88)*
High vs Low	6 (927)	1.10 (0.61, 2.00)	*P* = 0.001, *I*^2^ = 76.6%	1.12 (0.46, 2.69)
High vs Intermediate	6 (927)	0.21 (0.10, 0.45)	*P* = 0.000, *I*^2^ = 91.3%	0.08 (0.03, 0.18)
**Asian**				
*2 vs *1	7 (950)	0.41 (0.36, 0.46)	*P* = 0.915, *I*^2^ = 0.0%^#^	0.25 (0.21, 0.29)
*3 vs *1	7 (950)	0.45 (0.41, 0.51)	*P* = 0.451, *I*^2^ = 0.0%^#^	0.29 (0.24, 0.34)
*4 vs *1	3 (329)	0.05 (0.02, 0.16)	*P* = 0.011, *I*^2^ = 77.7%	0.04 (0.02, 0.06)
*3 vs *2	7 (950)	1.11 (0.97, 1.27)	*P* = 0.493, *I*^2^ = 0.0%^#^	1.16 (0.98, 1.38)
*4 vs *2	3 (329)	0.12 (0.04, 0.34)	*P* = 0.036, *I*^2^ = 69.9%	0.15 (0.09, 0.25)
*4 vs *3	3 (329)	0.09 (0.03, 0.26)	*P* = 0.032, *I*^2^ = 70.9%	0.13 (0.08, 0.21)
Intermediate vs Low	7 (1194)	2.59 (2.02, 3.31)*	*P* = 0.055, *I*^2^ = 51.3%	5.53 (2.12, 14.37)*
High vs Low	7 (1194)	0.87 (0.46, 1.62)	*P* = 0.000, *I*^2^ = 88.0%	0.89 (0.33, 2.39)
High vs Intermediate	7 (1194)	0.34 (0.18, 0.62)	*P* = 0.000, *I*^2^ = 90.5%	0.16 (0.06, 0.43)
*Results with significant differences; ^#^Low heterogeneity.			

For the Caucasian population, a significant cancer risk from UGTA7*3 vs *2 (pairwise: 1.55, 1.37 to 1.75; network: 1.94, 1.47 to 2.55) and Intermediate vs Low (pairwise: 4.98, 3.56 to 6.95; network: 14.79, 6.27 to 34.88) was found. In addition, for the Asian population, a significant cancer risk from Intermediate vs Low (pairwise: 2.59, 2.03 to 3.31; network: 5.53, 2.12 to 14.37) was identified.

## DISCUSSION

The network meta-analysis represents the most comprehensive synthesis of data for currently available data for UGT1A7 polymorphisms and gastrointestinal cancer risk. Direct and indirect trials comparing UDT1A7 polymorphic alleles and categorized UGT1A7 genotypes that were reported for 7738 participants were reviewed. First, it was found that compared with non-cancer participants, UGT*1 was significantly decreased in cancer patient groups, especially in the HCC, CRC, and Asian population groups; UGT*2 was significantly increased in the HCC and Asian population groups; UGT*3 was significantly increased in the HCC, CRC, Caucasian, and Asian population groups. Secondly, the UGT1A7 polymorphism alleles contrast model and the categorized UGT 1A7 genotypes were compared, and the outcomes revealed that the increased ratio of UGT*3 vs *2 may indicate an increased risk of cancer, especially in the PC and Caucasian groups. The ratio of Intermediate vs Low increased as well, which may also indicate an increased risk of cancer.

In recent years, the relation between gene polymorphisms, such as EGF, NAT2, DPYD, and UGTlAl, and the risk of GIC have attracted considerable attention. The UGT enzyme belongs to the super-gene family, which are the most important II metabolic enzymes in the human body. The catalytic glucoside reaction can catalyze alcohol, phenol, hydroxylamine, carboxylic acid, amides, mercaptan, and other types of chemical toxicities of the glucuronic acid binding reaction inactivation, and the detoxification mechanism plays an important role. UGTlA7 is a subtype of UGT, which changes metabolic enzyme activity due to mutations in genotypes and affects its ability to carry out detoxification, thereby altering the susceptibility of the body to carcinogenic factors [36].

This study extends the findings from primary case-controlled trials and previous meta-analyses by systematically synthesizing the efficacy data [19–35]. The meta-analysis differs from those in earlier studies in several ways. First, the main objective of the study was to determine the relationship between UGT1A7 gene polymorphisms and the risk of gastrointestinal cancer, whereas the previous pairwise studies included all cancer-related publications [17–18]. Secondly, subgroup analyses and meta-regressions were used to identify the differences between the different cancer types and ethnicities to determine which population has a higher risk of UGT1A7 gene polymorphisms. Finally, a network meta-analysis was used to directly and indirectly compare the UGT1A7 polymorphism alleles contrast model and the categorized UGT 1A7 genotypes.

**Figure 3:**
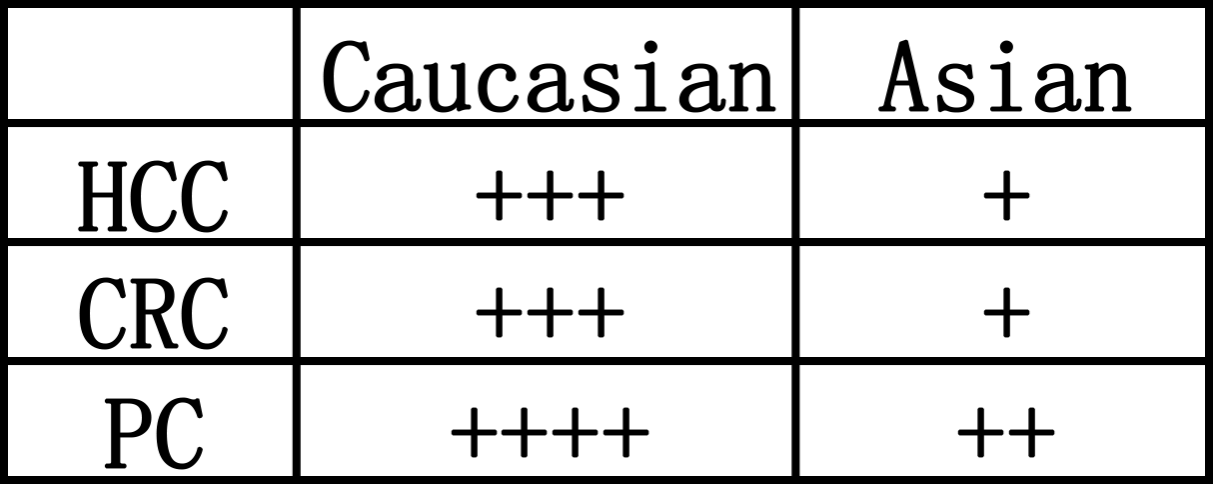
Summary of cancer risk associated with UGT1A7 *3vs*2. A + means one significant result

This review followed the guidelines for conducting rigorous systematic reviews and network meta-analyses [39–41]. To identify as many relevant reports as possible and to decrease the risk of bias, a comprehensive search strategy was designed. Based on these considerations, little evidence of publication bias was observed during the statistical assessment (Table 2). The increased ratio of UGT1A7*3 vs *2 may indicate an increased risk of GIC (Table 3). Figure 3 summarizes the cancer risks associated with UGT1A7 *3 vs *2. A + means one significant result. It was observed that the sensitivity of UGT1A7 *3 vs *2 was highest in the Caucasian population with PC, followed by the Caucasian population with HCC and CRC. The Asian population with HCC and CRC ranked lowest, followed by the Asian population with PC. Moreover, the increased ratio of the categorized UGT 1A7 genotypes for Intermediate vs Low revealed a higher GIC risk for all cancer types and ethnicities.

These results were proposed by the meta-analysis for the first time, and there is no published evidence to support this finding; however, it is logical to assume that a mutant type *3 with decreasing enzyme activity (compared with type*1 wild type) vs a mutant type *2 with a similar enzyme activity could increase cancer risks. Each time a normal cell divides, several errors or mutations occur. Most of these mutations do not cause harm because they occur in junk DNA, in genes unrelated to cancer, or in unimportant areas. Therefore, not all mutant-type genes are harmful. Though this is usually the case, which is favorable, this can cause harm when cancer-driven genes are involved [37]. It is suggested that the UGT1A7 *2 mutation is a cancer suppressor in the Caucasian population, while it promotes cancer in the Asian population, and the UGT1A7 *3 mutation is cancer-promoting.

The network meta-analysis had some limitations that merit further discussion. First, in the GRADE framework, several comparisons were determined to be moderate or low-quality, which largely restricts the interpretation of the results. In addition, the network analysis contained some inconsistencies, which were mainly determined by the loop. Moreover, only four alleles were measured, and some alleles (such as UGT1A7 *5–*11) were not analyzed due to the small sample size. Furthermore, positive results are likely to be published, while negative results are not likely to be shared. An additional limitation of the pairwise outcomes was the extensive heterogeneity (Tables 2, 3), which indicated a substantial variability in the outcomes of the included studies, though this was often due to the presence of heterogeneity in the baseline outcomes (Table 1) and the differences observed in the cancer types and populations. Finally, different genotypic methods (RFLP-PCR and PCR) could also lead to differences in resulets. Ways to reduce the risk of bias include defining the groupings of original studies, providing each patient’s data, and expanding the scope of the studies to a global scale. In the included studies, randomization and blinding were not performed, and the quality of the studies was low (Supplementary Table 4). The studies’ levels of quality were only sufficient for a meta-analysis. Further case-controlled studies of UGT1A7 gene polymorphisms should have a large sample size and should be robust and randomized to confirm the risks of cancer due to genotypes, particularly in patients with GIC. Additional normative studies should be conducted for future network meta-analyses.

The findings of this comprehensive network meta-analysis provide some evidence that there are cancer risks associated with increased UGT1A7 *2 for the HCC and Asian groups and with increased UGT1A7 *3 for the HCC, CRC, Caucasian, and Asian groups. Moreover, in Caucasian patients with GIC, the ratio of UGT1A7 *3 vs *2 was increased. Genotype studies revealed that increased UGT1A7* 3 vs *2 ratios were the most likely to result in GIC, especially in the Caucasian population. In a clinical setting, the genetic monitoring of UGT1A7 can be used as a predictor of cancer.

## MATERIALS AND METHODS

### Search strategy and selection criteria

This systematic review was performed with an a priori established protocol (PROSPERO CRD42017064826) [38], and the meta-analysis was performed in agreement with the preferred reporting items for the systematic reviews and meta-analyses (PRISMA) statement, the PRISMA network statement, and the Cochrane Collaboration recommendations [39–41]. For this network meta-analysis, PubMed, Embase, and the Cochrane Central Register (see Supplementary Table 2 for more details) were searched for case-controlled trials (CCT) published from the date of database inception to May 2017 that compared UGT 1A7 gene polymorphism genotypes in gastrointestinal cancer patients with non-cancer control groups. No restrictions were placed on language.

The inclusion criteria consisted of: case-controlled trials of subjects with and without gastrointestinal carcinomas (such as hepatocellular carcinoma, colorectal cancer, etc.); participants of any age, gender, tumor stage, and histological grade; either Caucasian or Asian; and either using PCR as a genotypic method or DNA sequencing for detection. The exclusion criteria were: background records of comparing UGT 1A7 gene polymorphism genotypes in gastrointestinal cancers and an inappropriate control in the studies. Moreover, studies that used inappropriate tumor types (non-GIC) and that had no specific data, no human subjects, and no usable data were excluded.

### Data abstraction and assessment of the risk of bias

Three researchers (ZYS, HJ, and FF) independently extracted data on studies, participants, and genotype-related characteristics to a standardized form, and discrepancies were resolved by consensus by referring to the original study in consultation with a fourth reviewer (LBA or ZQC). Data on UGT 1A7 gene polymorphism genotypes were extracted from original studies. Cancer type, trial size, and details of genetic polymorphisms, including UGT1A7 allele frequency and UGT1A7 categorized genotype, were also extracted.

To reduce the risk of bias of individual studies, the Newcastle-Ottawa scale score [42] was used as a tool to evaluate the methodological quality. The scale is based on the Newcastle-Ottawa scale’s “yes” or “no” answers to the following criteria: (1) Is the case definition adequate? (2) Is there representativeness of the cases? (3) Is there selection of controls? (4) Is there a definition of controls? (5) Is there comparability of cases and controls? (6) Is there ascertainment of exposure? (7) Is the same method of ascertainment used for cases and controls? (8) Is there a non-response rate? A system analysis of studies was performed that excluded those with scores less than 5. The risk of bias assessments were performed independently by two investigators and were resolved by a third researcher when needed.

### Outcomes

Subgroup analyses and a meta-regression were performed according to cancer type (HCC, CRC, and PC) and ethnicity (Caucasian, Asian, and African Americans).

The primary outcome was that a gastrointestinal cancer risk was associated with UGT1A7 polymorphism alleles (*1, *2, *3 and *4), which could be stratified by cancer type and ethnicity. The secondary outcome was the UGT1A7 polymorphism alleles contrast model and UGT 1A7 genotypes categorized according to enzymatic activity (High, Intermediate, and Low), which could also be stratified by cancer type and ethnicity.

### Data synthesis and statistical analysis

Studies that reported multiple cancer types and ethnicities were categorized as sub-studies (marked as a/b) to avoid double counting and mistreating data. First, a direct meta-analysis was performed using a random effects model because it is likely the most appropriate and conservative methodology to account for between-trial heterogeneity within each comparison [43–44]. To estimate pooled odds ratios (OR) and 95% confidence intervals incorporating heterogeneity within and between studies, STATA v14.0 was used. Statistical heterogeneity was assessed with a *P* value and *I*^*2*^ statistic, with values over 50% indicating substantial heterogeneity [45]. The Begg’s and Egger’s tests were used to detect publication bias [46].

Second, a random-effects network meta-analysis was conducted using STATA v14.0. The results of the network meta-analysis were summarized using OR and their credible intervals (CrI) [47]. A common heterogeneity parameter was assumed for all comparisons, and the global heterogeneity was assessed using the *P* value and the *I*^*2*^ statistic.

Each relative UGT1A7 polymorphism allele and UGT1A7 genotype resulted from the combination of the direct and the indirect evidence derived from the network meta-analysis, which was assumed to be coherent [43]. Inconsistencies between direct and indirect sources of evidence were statistically assessed globally (by comparison of the fit and parsimony of consistency and inconsistency models) and locally (by calculating the difference between the direct and indirect estimates in all closed loops in the network) [48]. When a direct connection between two treatment arms was not available, the results were based on indirect evidence.

### Quality of evidence

In addition, the quality of evidence for the primary outcomes was assessed based on the GRADE system using GRADEpro GDT [49–50]. The GRADE system assesses risk of bias (study limitations), imprecision, inconsistency, indirectness of study results, and publication bias (classifying each as high, moderate, low, or very low) across the body of evidence to derive an overall summary of the quality of evidence.

### Patient involvement

No patients were involved in forming the research question or the outcome measures, nor were they involved in developing plans for the design or the implementation of the study. No patients were asked to advise in the interpretation or writing of the results. There is no intention to disseminate the results of the research to the study participants or to the relevant patient community.

## SUPPLEMENTARY MATERIALS FIGURE AND TABLES




