# Supplementary material for: Genetic polymorphisms in human UDP-glucuronosyltransferases 1A7 and the risk of gastrointestinal carcinomas: A systematic review and network meta-analysis
Source: Oncotarget. 2017 Jun 27;8(39):66371–81. doi: 10.18632/oncotarget.18675 (PMC5630419; doi:10.18632/oncotarget.18675)
Supplement: Supplementary file 1 [file oncotarget-08-66371-s001.pdf]

Genetic polymorphisms in human UDP-glucuronosyltransferases 1A7 and the risk of gastrointestinal carcinomas: A systematic review and network meta-analysis

Supplementary Materials

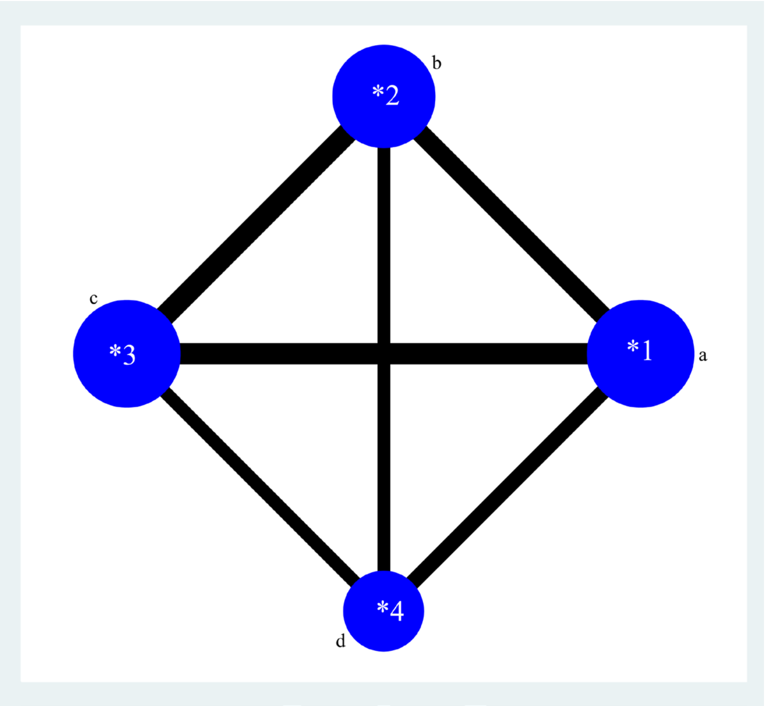

Supplementary Figure 1: Network of eligible comparisons for UGT1A7 polymorphism alleles (*1, *2, *3 and *4).

Supplementary Table 1: The UGT1A7 polymorphic variants and categorized genotypes according to the enzymatic activity in systematic review meta-analysis

UGT1A7	*1	*2	*3	*4
Amino acid change	$N^{129}R^{131}W^{208}$	$K^{129}K^{131}W^{208}$	$K^{129}K^{131}R^{208}$	$N^{129}R^{131}R^{208}$
Functional change	Wild type	Similar activity	Decreased activity	Decreased activity
Categorized UGT1A7 genotypes		High	Intermediate	Low
		*1/*1	*1/*2, *1/*3, *1/*4, *2/*2, *2/3, *2/*4	*3/*3, *3/*4, *4/*4

Supplementary Table 2: Study characteristic. See Supplementary_Table_2

Supplementary Table 3: Risk of bias assessments

Low Risk of Bias	(green)
Intermediate or Unknown Risk of Bias	(yellow)
High Risk of Bias	(red)

Study	Is the case definition adequate	Representativeness of the cases	Selection of Controls	Definition of Controls	Comparability of cases and controls (/2)	Ascertainment of exposure(/2)	Same method of ascertainment for cases and controls	Non-Response rate	Overall rating and TOTAL SCORE / 10
Borentain P, 2007 [19]	1	1	1	1	2	1	1	1	9
Jia ZY, 2010 [20]	1	1	1	1	2	0	1	1	8
Kong SY, 2008 [21]	1	1	1	1	2	1	1	1	9
Stücker I, 2007 [22]	1	1	1	1	2	1	1	1	9
Tseng CS, 2005 [23]	1	1	1	1	2	0	1	1	8
Vogel A, 2001 [24]	1	1	1	1	2	0	1	1	8
Wang Y, 2004 [25]	1	1	1	1	2	1	1	1	9
Butler LM(a), 2005 [26]	1	1	1	1	2	1	1	1	9
Butler LM(b), 2005 [27]	1	1	1	1	2	1	1	1	9
Chen K, 2006 [28]	1	1	1	1	2	1	1	1	9
Osawa K, 2012 [29]	1	1	1	1	2	1	1	1	9
Strassburg CP, 2002 [30]	1	1	1	1	2	1	1	1	9
Tang KS, 2005 [31]	1	1	1	1	2	0	1	1	8
Van der Logt EM, 2004 [13]	1	1	1	1	2	0	1	1	8
Ockenga J, 2003 [32]	1	1	1	1	2	0	1	1	8
Piepoli A, 2006 [33]	1	1	1	1	2	0	1	1	8
Verlaan M, 2005 [34]	1	1	1	1	2	1	1	1	9
Vogel A, 2002 [35]	1	1	1	1	2	1	1	1	9

Risk of Bias Assessment using the Newcastle-Ottawa Scale for Case-control Studies.

Supplementary Table 4: Search strategies for Pubmed, EMBASE and the cochrane library database

Search strategies for PubMed

#1. (“glucuronosyltransferase”[MeSH Terms] OR “glucuronosyltransferase”[All Fields] OR (“udp”[All Fields] AND “glucuronosyltransferase”[All Fields]) OR “udp glucuronosyltransferase”[All Fields]) AND (“genes”[MeSH Terms] OR “genes”[All Fields] OR “gene”[All Fields]) AND (“polymorphism, genetic”[MeSH Terms] OR (“polymorphism”[All Fields] AND “genetic”[All Fields]) OR “genetic polymorphism”[All Fields] OR “polymorphism”[All Fields]) AND (“genotype”[MeSH Terms] OR “genotype”[All Fields]) AND (“neoplasms”[MeSH Terms] OR “neoplasms”[All Fields] OR “cancer”[All Fields])

#2. agt[All Fields] AND (“genes”[MeSH Terms] OR “genes”[All Fields] OR “gene”[All Fields]) AND (“polymorphism, genetic”[MeSH Terms] OR (“polymorphism”[All Fields] AND “genetic”[All Fields]) OR “genetic polymorphism”[All Fields] OR “polymorphisms”[All Fields]) AND (“genotype”[MeSH Terms] OR “genotype”[All Fields]) AND (“neoplasms”[MeSH Terms] OR “neoplasms”[All Fields] OR “cancer”[All Fields])

#3. 1 and 2

#4.(“glucuronosyltransferase”[MeSH Terms] OR “glucuronosyltransferase”[All Fields] OR (“udp”[All Fields] AND “glucuronosyltransferase”[All Fields]) OR “udp glucuronosyltransferase”[All Fields]) AND 1a7[All Fields] AND (“carcinoma, hepatocellular”[MeSH Terms] OR (“carcinoma”[All Fields] AND “hepatocellular”[All Fields]) OR “hepatocellular carcinoma”[All Fields] OR (“hepatocellular”[All Fields] AND “carcinoma”[All Fields]))

#5. UGT[All Fields] AND 1A7[All Fields] AND (“carcinoma, hepatocellular”[MeSH Terms] OR (“carcinoma”[All Fields] AND “hepatocellular”[All Fields]) OR “hepatocellular carcinoma”[All Fields] OR (“hepatocellular”[All Fields] AND “carcinoma”[All Fields]))

#6.4 and 5

#7.(“glucuronosyltransferase”[MeSH Terms] OR “glucuronosyltransferase”[All Fields] OR (“udp”[All Fields] AND “glucuronosyltransferase”[All Fields]) OR “udp glucuronosyltransferase”[All Fields]) AND 1a7[All Fields] AND (“colorectal neoplasms”[MeSH Terms] OR (“colorectal”[All Fields] AND “neoplasms”[All Fields]) OR “colorectal neoplasms”[All Fields] OR (“colorectal”[All Fields] AND “carcinoma”[All Fields]) OR “colorectal carcinoma”[All Fields])

#8. UGT[All Fields] AND 1A7[All Fields] AND (“colorectal neoplasms”[MeSH Terms] OR (“colorectal”[All Fields] AND “neoplasms”[All Fields]) OR “colorectal neoplasms”[All Fields] OR (“colorectal”[All Fields] AND “carcinoma”[All Fields]) OR “colorectal carcinoma”[All Fields])

#9.7 and 8

#10.1A7[All Fields] AND Pancreatic[All Fields] AND carcinoma[All Fields]

#11.UGT[All Fields] AND 1A7[All Fields] AND Pancreatic[All Fields] AND carcinoma[All Fields]

#12. 11and 12

#13.1A7[All Fields] AND Gastrointestinal[All Fields] AND (“carcinoma”[MeSH Terms] OR “carcinoma”[All Fields] OR “carcinomas”[All Fields])

#14.UGT[All Fields] AND 1A7[All Fields] AND Gastrointestinal[All Fields] AND (“carcinoma”[MeSH Terms] OR “carcinoma”[All Fields] OR “carcinomas”[All Fields])

#15. 13 and 14

#16. 6 OR 9 OR 12 OR 15

Search strategies for EMbase

#1 ‘UDP—glucuronosyhransferase$’:ab,ti

#2 ‘UGT$’:ab,ti

#3 ‘gene polymorphisms genetype 1A7$’:ab,ti

#4 ‘Hepatocellular carcinoma$’/exp

#5 ‘Colorectal carcinoma$’:ab,ti

#6 ‘Pancreatic carcinoma’:ab,ti

#7 ‘Gastrointestinal carcinoma’:ab,ti

#8 #1 OR #2 OR #3 OR #4 OR #5 OR #6 OR #7

Search strategies for Cochrane library

#1.UDP—glucuronosyhransferase and UGT, Hepatocellular carcinoma or Colorectal carcinoma or Pancreatic carcinoma or Gastrointestinal carcinoma.

#2. MeSH descriptor.
