# Supplementary material for: Genetic polymorphisms in human UDP-glucuronosyltransferases 1A7 and the risk of gastrointestinal carcinomas: A systematic review and network meta-analysis
Source: Oncotarget. 2017 Jun 27;8(39):66371–81. doi: 10.18632/oncotarget.18675 (PMC5630419; doi:10.18632/oncotarget.18675)
Supplement: Supplementary file 2 [file oncotarget-08-66371-s002.doc]

**Supplementary Table 2: S**tudy characteristic

| Publication,year | Cancer types | Total (Case/Control) | Ethnicity, Location | Design | Genotyping method | UGT1A7 allele frequency (Case/Control) | | | | UGT1A7 categorised genotype (Case/Control) | | |
| --- | --- | --- | --- | --- | --- | --- | --- | --- | --- | --- | --- | --- |
|  |  |  |  |  |  | *1 | *2 | *3 | *4 | High | Intermediate | Low |
| Borentain P , 2007[19] | HCC | 56/77 | Caucasian, France | Randomly, case-controlled | ARMS-PCR and RFLP-PCR | 38/57 | 27/31 | 47/63 | 0/3 | 6/14 | 41/47 | 9/16 |
| Jia ZY, 2010[20] | HCC | 136/136 | Asian, China | Case-controlled | AS-PCR and RFLP-PCR | 145/179 | 61/47 | 66/46 | - | 41/62 | 63/55 | 32/19 |
| Kong SY, 2008[21] | HCC | 244/314 | Asian, Korea | Case-controlled | PCR and DNA sequencing | 264/372 | 102/98 | 122/158 | - | 69/106 | 124/156 | 51/52 |
| Stücker I, 2007[22] | HCC | 164/131 | Caucasian,France | Randomly, case-controlled | PCR | 138/87 | 74/73 | 116/102 | - | 71/48 | 70/64 | 23/19 |
| Tseng CS, 2005[23] | HCC | 217/291 | Asian, China | Case-controlled | RFLP-PCR and DNA sequencing | 236/388 | 106/109 | 92/85 | - | 64/144 | 108/120 | 45/37 |
| Vogel A, 2001[24] | HCC | 59/70 | Caucasian, German | Case-controlled | PCR, DNA sequencing and TGGE | 38/79 | 45/38 | 52/22 | 3/5 | 4/29 | 46/33 | 9/8 |
| Wang Y, 2004[25] | HCC | 122/158 | Asian, Japan | Case-controlled | PCR and DNA sequencing | 127/208 | 46/44 | 69/54 | 2/10 | 36/73 | 55/62 | 31/23 |
| Butler LM(a), 2005[26] | CRC | 197/202 | African Americans, USA | Randomly, case-controlled | PCR and DNA sequencing | 146/150 | 149/158 | 98/96 | 1/0 | 24/25 | 161/167 | 12/10 |
| Butler LM(b), 2005[27] | CRC | 203/210 | Caucasian,France,USA | Randomly, case-controlled | PCR and DNA sequencing | 142/132 | 106/119 | 158/168 | 0/1 | 26/26 | 142/149 | 35/35 |
| Chen K, 2006[28] | CRC | 140/280 | Asian, China | Randomly, case-controlled | SN-PCR, AS-PCR and RFLP-PCR | 140/344 | 63/75 | 71/132 | 6/9 | 40/104 | 80/159 | 20/17 |
| Osawa K, 2012[29] | CRC | 67/112 | Asian, Japan | Case-controlled | RFLP-PCR | 60/138 | 28/39 | 38/45 | 8/2 | 1/32 | 58/76 | 8/4 |
| Strassburg CP, 2002[30] | CRC | 78/210 | Caucasian, German | Case-controlled | PCR and TGGE | 57/191 | 36/121 | 47/73 | 16/35 | 7/42 | 62/151 | 9/17 |
| Tang KS, 2005[31] | CRC | 268/441 | Asian, China | Case-controlled | RFLP-PCR and DNA sequencing | 302/560 | 114/193 | 120/129 | - | 76/184 | 182/246 | 10/11 |
| Van der Logt EM, 2004[13] | CRC | 367/405 | Caucasian,Netherland | Case-controlled | Case-controlled | 238/275 | 194/204 | 300/321 | - | 35/51 | 271/279 | 60/70 |
| Ockenga J, 2003[32] | PC | 52/235 | Caucasian, German | Case-controlled | PCR and DNA sequencing | 39/207 | 21/134 | 35/96 | 9/33 | - |  |  |
| Piepoli A, 2006[33] | PC | 61/105 | Caucasian, Italy | Case-controlled | RFLP-PCR | 54/74 | 15/46 | 52/89 | 1/1 | 10/14 | 40/74 | 11/17 |
| Verlaan M, 2005[34] | PC | 236/1409 | Caucasian,Czech Republic+German+Netherland+Switzerland | Randomly, case-controlled | FRET and RFLP-PCR | 149/979 | 123/642 | 184/1135 | - | 26/179 | 169/953 | 33/246 |
| Vogel A, 2002[35] | Proximal digestive tract cancer | 76/210 | Caucasian, German | Case-controlled | PCR, DNA sequencing and TGGE | 66/182 | 24/126 | 49/80 | 13/32 | - |  |  |

PCR, Polymerase Chain Reaction; ARMS-PCR, Amplification Refractory Mutation System (ARMS) - PCR test; PCR-RFLP, PCR-Restriction Fragment Length Polymorphism; allele-specific PCR (AS-PCR); TGGE, temperature gradient gel electrophoresis; SN-PCR, seminested PCR; FRET, fluorescence resonance energy transfer;
